# Women’s Health: Does City Life Alter Breast Density?

**DOI:** 10.1289/ehp.116-a66

**Published:** 2008-02

**Authors:** M. Nathaniel Mead

Epidemiologic studies have found differences between urban and rural women in breast cancer incidence and mortality, both generally being higher in urban areas. Now a British study suggests that women who live or work in urban areas have denser breasts and thus potentially a greater risk of breast cancer, according to a report published online 19 December 2007 ahead of print in *Current Medical Research and Opinion*. The new findings add to the evidence that breast cancer risk is higher in urban settings and may eventuate in new breast cancer screening guidelines.

Breasts are made up of glandular and fatty tissue. Glandular tissue is denser than fatty tissue and harder to read on a mammogram; a higher percentage of dense tissue is also linked with increased risk of breast cancer. A meta-analysis of more than 40 studies published in the June 2006 issue of *Cancer Epidemiology, Biomarkers & Prevention* concluded that women with high breast density have a nearly 5-fold higher breast cancer risk than women with the lowest breast density.

The researchers analyzed digital mammograms of 972 women, including 318 women from London and 654 women from outside the capital. All the women had received mammography at The Princess Grace Hospital in London. The researchers found that women aged 45–54 who lived in central London were twice as likely to have very dense breasts as women who lived in outlying suburban and rural areas. Age-specific analyses indicated that breast density differences by area were more pronounced in women under age 50.

Study leader Nicholas M. Perry, director of the London Breast Institute at The Princess Grace Hospital, was not particularly surprised by the study’s findings. While working as a radiologist over a 15-year period, Perry had observed that London women tended to have denser breasts than women living in outlying areas. These personal observations were the main impetus for the study. “Our study was not designed to do anything other than report on the original observation that urban women seemed to have denser breasts,” he says. “Since releasing our findings, I have received numerous comments from radiologists who have observed the same phenomenon.”

The biological basis for the new finding remains to be determined. “It may simply be that London women are thinner than women living outside the capital, or it may be due to other factors,” says coauthor Stephen Duffy, an epidemiologist at Cancer Research UK and Queen Mary, University of London. “We need to carry out research on density and other breast cancer risk factors at the individual level by area of residence to get to the bottom of this.”

Although mammographic density is a strong risk factor for breast cancer, it is not the only one. As an observational study, the London study did not control for factors such as suboptimal body weight, which also can influence density and is an independent risk factor for breast cancer.

“Before it can be concluded that urban or rural residence influences mammographic density, it must be shown that the individuals living in urban or rural environments do not differ in one or more factors that influence density,” says Norman Boyd, a senior scientist at the Campbell Family Institute for Breast Cancer Research at Princess Margaret Hospital in Toronto. “Body weight is a strong influence on density, and it is known that obesity is more common in rural than in urban settings, and that obesity is less common among professionals than in those with other occupations. Both of these factors could explain the observed findings. If those in rural settings also had more children, that could also contribute.” Boyd’s research team is presently focused on identifying the genetic and environmental factors that influence mammographic density and the relationship of these factors to breast cancer risk.

Perry’s team speculates that their observations can be at least partially attributed to environmental factors such as estrogenic particles present in traffic emissions. They present this argument in a letter to the editor published online 8 December 2007 ahead of print in *Cancer Causes & Control*. Still, Perry cautions that more research is needed to determine the underlying reasons for this phenomenon, taking into account stress as well as lifestyle and environmental factors. “We intend to carry on with this research in the form of an intervention trial and hope other groups will do the same,” he says. “There is also likely to be a consensus conference before long addressing the issue of breast density and how it is modulated.”

In the interim, he urges urban women to be especially vigilant about breast cancer screening and to rely primarily on digital mammography, which is more effective than conventional mammography at detecting cancer in dense breast tissue. At the present time, only about 25% of centers in the United States and 10% of mammography centers in England offer digital mammography.

## Figures and Tables

**Figure:**
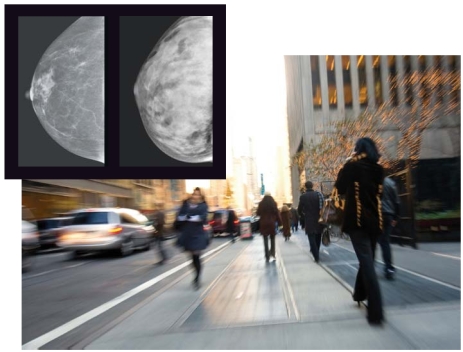
Population density? Observational data suggest city women have denser breast tissue than women in outlying areas, but the reason for the difference is unclear (inset, l–r: less dense and more dense breast tissue).

